# Prevalence of Cognitive Impairment and Depression among a Population Aged over 60 Years in the Metropolitan Area of Guadalajara, Mexico

**DOI:** 10.1155/2012/175019

**Published:** 2012-12-03

**Authors:** Genaro G. Ortiz, Elva D. Arias-Merino, María E. Flores-Saiffe, Irma E. Velázquez-Brizuela, Miguel A. Macías-Islas, Fermín P. Pacheco-Moisés

**Affiliations:** ^1^Laboratorio Desarrollo-Envejecimiento, Enfermedades Neurodegenerativas, Centro de Investigación Biomédica de Occidente (CIBO), Instituto Mexicano del Seguro Social (IMSS), Sierra Mojada 800, 44340 Guadalajara, JAL, Mexico; ^2^Departamento de Salud Pública, Centro Universitario de Ciencias de la Salud (CUCS), Universidad de Guadalajara, 44350 Guadalajara, JAL, Mexico; ^3^OPD-Instituto Jalisciense de Cancerología JAL, Guadalajara, JAL, Mexico; ^4^Departamento de Neurología, Unidad Médica de Alta Especialidad (UMAE), Centro Médico Nacional de Occidente (CMNO), IMSS, 44340 Guadalajara, JAL, Mexico; ^5^Departamento de Química, Centro Universitario de Ciencias Exactas e Ingenierías, Universidad de Guadalajara, Boulevard Marcelino García Barragán 1421, 44430 Guadalajara, JAL, Mexico

## Abstract

*Background*. Cognitive impairment is an important clinical issue among elderly patients with depression and has a more complex etiology because of the variable rate of neurodegenerative changes associated with depression. The aim of the present work was to examine the prevalence of cognitive impairment and depression in a representative sample of adults aged ≥60 years. *Methods*. The presented work was a cross-sectional study on the prevalence of cognitive impairment and depression. Door-to-door interview technique was assigned in condition with multistage probability random sampling to obtain subjects that represent a population of the Guadalajara metropolitan area (GMA), Mexico. Cognitive function and depression were assessed by applying standardized Mini-Mental State Examination of Folstein (MMSE) and the Geriatric Depression Scale (GDS), respectively. *Results*. Prevalence of cognitive impairment was 13.8% (14.5% women, 12.6% men); no significant differences by gender and retired or pensioner were found. Prevalence of depression was 29.1% (33.6% women, 21.1% men); no significant differences by retired or pensioner were found. Cognitive impairment was associated with depression (OR  =  3.26, CI 95%, 2.31–4.60). Prevalence of cognitive impairment and depression is associated with: being woman, only in depression being older than 75 years being married, and a low level of education. *Conclusion*. Cognitive impairment and depression are highly correlated in adults aged ≥60.

## 1. Introduction

As our society ages, age-related diseases assume increasing prominence as both personal and public health concerns [1]. In Mexico, the annual growth rate of the elderly population was 3.5% in 2000, which if maintained, the current older-adult population (7.6%) would double every 19 years and would amount to 28% of the total Mexican population in 2050 [2]. Late life depression occurring in patients over age 65 is a serious illness and may lead to impaired physical function, increased mortality, and unwarranted use of health care resources. Depression in older adults remains under-diagnosed and under-treated. The prevalence of depression varies depending on the population studied, affecting up to 9% of community dwelling elderly but 25% of institutionalized elderly and those recently hospitalized [1–3]. Patients with depression often present with cognitive complaints or cognitive deficits. These cognitive changes may occur as a consequence of depression or may indicate a coexisting condition such as Alzheimer's disease or Parkinson's disease. Recent research on the prevalence of cognitive impairment in elderly show considerable variability with estimates ranging from 4 to 5% to 40% or more. This variability depends on different diagnostic criteria used, the degree of severity of clinical manifestations, and the age range used in the study, among other factors [4–6].

In Mexico, little is known about the prevalence of cognitive impairment and depression in older adults. For instance, the few depression prevalence studies carried out have rarely focused on persons 60 years and older, and, consequently, sample sizes have been small to yield precise estimates [7–10]. That knowledge is important to get items to help plan health services to this population, so the aim of this work is to estimate the prevalence of cognitive impairment and depression among the population 60 years and older who resides in the Guadalajara metropolitan area, Mexico.

## 2. Methods

This is a cross-sectional, descriptive, and transversal study. The study was conducted in the Guadalajara metropolitan area (GMA). The GMA is the second largest city (Mexico) in demographic terms; it includes the core municipality of Guadalajara and the surrounding municipalities of Zapopan, Tlaquepaque, Tonalá, El Salto, Tlaquepaque, and Zapotlanejo. The six municipalities of GMA are subdivided into 14 urban basic geostatistical areas (UGEA). We used multistage and proportional random sampling to obtain our study sample of adults. The sample size obtained was 1142 adults. Within the 14 UGEAs, we conducted the random selection of blocks. Located the block, we proceed at the southwest corner clockwise until we find an adult 60 years or more. Adults 60 years or more living at least one year at the GAM were invited to participate. Data were collected by means of structured personal interviews conducted by trained interviewers. All participants or family members provided informed consent. Sociodemographic data (age, gender, marital status, education, and occupation) were obtained from the first interview. 

 The institutional review board of the Instituto Mexicano del Seguro Social approved the study protocol. 

Cognitive function was assessed by applying standardized Mini-Mental State Examination (MMSE) of Folstein [11]. MMSE scores range from 0 to 30, with lower scores indicating increasing severity of cognitive impairments in the domains of orientation, memory, attention, and executive functions. Subjects with cognitive impairment had scores between 0 and 18. The sensitivity was 87%, and specificity was 82%. 

Depression was assessed with the Geriatric Depression Scale (GDS) [12], a questionnaire specifically developed for screening depressive symptoms in elderly populations. The cutoff for normal range were 10. The sensitivity and specificity was 84% and 95%, respectively.

### 2.1. Statistics

The data obtained were analyzed with the statistical package for social sciences software (SPSS). Data inconsistencies were corrected, and some data were transformed into dichotomous variables to fit the statistical analysis. The prevalence of cognitive impairment and depression was calculated in percent. Estimated odds ratios (ORs) crude and adjusted and corresponding 95% confidence intervals (CIs) were obtained.

## 3. Results 

1142 adults of both genders (413 men and 729 women) aged 60 to 110 years (mean age 71.6 ± 8.3 years) were evaluated. The average age for male and female was essentially the same (*P* = 0.27). Regarding marital status, 49.0% were married or living with a partner, and 51.0% were single; significant differences by gender were found (*P* = 0.000) as more women were not married (60.1%), and more men were married (70.5%). The schooling range was 0 to 23 years, and the average was 4.42 years (4.15 for women and 4.89 for men). No significant differences were observed in education years by gender (*P* = 0.09). It is noteworthy that 23.2% of the adults had no formal educational instruction. 73.9% of the sample did not have the benefit of retirement or have a pension, while 26.1% are retirees and pensioners (18.5% women; 39.5% men). Differences in the benefit of retirement or pension between men and women were found (see Table 1).

As shown in Table 2, the overall prevalence of cognitive impairment in this sample was 13.8% (CI 95%; 11.9–16.0). Women showed a higher proportion of cognitive impairment (14.5%, CI 95%; 12.1–17.4) than men (12.6%, CI 95%; 9.6–16.3), although no significant differences by gender were found (*P* = 0.37). The overall prevalence of depression was 29.1%. Women showed a higher proportion of depression (33.6%) than men (21.1%). 

Data analysis of odds ratio showed in Table 3 suggests that the prevalence of cognitive impairment is associated with being older than 75 years (OR = 4.92 CI 95%; 3.43–7.06), being not married (OR = 3.48 CI 95%; 2.39–5.08), low educational level (OR = 9.06 CI 95%; 5.16–4.60), and depression (OR = 3.26 CI 95%; 2.31–4.60). Likewise, exhibited in Table 3, demographic variables (being older than 75 years, being not married, and low educational level) and depression were associated with cognitive impairment in adjusted odd rate.

Data analysis between sociodemographic variables exhibited as depression is associated with being female (OR = 1.89 CI 95%; 1.43–2.51), being older than 75 years (OR = 1.32 CI 95%; 1.03–1.76), being not married (OR = 1.89 CI 95%; 1.46–2), low educational level (OR = 2.75 CI 95%; 2.07–3.65), and cognitive impairment (OR = 3.26 CI 95%; 2.31–4.60). Similarly we exhibited in Table 3, demographic variables that being woman, being not married, low educational level, and cognitive impairment were associated with depression in adjusted odd rate (Table 4).

## 4. Discussion 

The data obtained in this work shows that the overall prevalence of cognitive impairment in a representative sample of older adults of the GMA was 13.8%. This result is higher than the obtained previously in the whole Mexican population that was 7.7% [10]. This difference with the data from the present work could be explained by the allocation of subjects, variety of instruments (self-report scale and interviewer's rating scale), staff (psychiatric and nonpsychiatric), type of sample, and other factors which are specific characteristics of the population of the GAM. Interestingly, risk factors for cognitive impairment detected in this work were being older than 75 years, being woman, single, retired, or pensioned, and low scholarship.

The overall prevalence of depression found in this work was 29.1%. This data was lower than the previously reported [13], which found a prevalence of 36.2%. That discrepancy may be due to the number and characteristics of the sample. Prevalence studies in Europe, the United States, and Canada reveal relatively consistent findings. For instance, 22.2% of individuals in the United States of age 71 years or older have cognitive impairment without dementia [14], and Canadian samples aged 65 and over report prevalence rates for cognitive impairment without dementia of 16.8% [15]. In contrast, the prevalence of depression in elderly New Mexico Hispanic population was 13.2% (6.4% in men and 16.9% in women) [16].

In consonance with the previously reported data [17], the prevalence of depression increases with the age. The elderly may have chronic-degenerative or neurological illness more often than other age groups as suggested by Katon and Sullivan [18] In addition, social characteristics were important factors that affect depression such as retirement, loss of a partner, or loss of friends.

The prevalence of depression was higher in women than in men and is correlated with the sociodemographic data analyzed (age, being married, low scholarship, and being not retired or pensionated). These differences of gender have too many depression-related phenomena. Therefore, it is not well understood but probably related to a combination of biological and genetic factors including hormonal changes as well as from the stress from working life, family responsibility, and social roles [19].

For both conditions (cognitive impairment and depression), the probability is higher in women and increases with age, being married, a low scholarship, and subjects without pension or not retired. According to the present data, most epidemiological studies have shown an association between low education and cognitive impairment [20]. In this work, 74% of older adults do not have retirement or pensions, and most of these are lacking a family, and institutional or social support. This may create a situation of loneliness and isolation affectively and loss of roles that can lead to depression, which plays an important role for cognitive impairment. 

We found similar prevalence of depression compared with the study made in Dublin [21]. It is worthy to note that as reported in the Mexico-American population, there is a link between depression and symptoms associated with cognitive impairment [22, 23], and this study found OR = 3.75 (CI 95% 2.63–5.34). The reported prevalence of cognitive impairment in Cuba varies from 4.2% to 19.6% this increases to more age [24], and similar data was obtained in our study. Other authors also confirm that depressive symptoms are associated with cognitive impairment [25], but by itself, mild depression does not lead to a severe cognitive impairment [26], but the most severe cases of chronic depression did so significantly [25]. Indeed, mild cognitive impairment among elderly population, which is determined by multiple etiologies, signifies and amplifies the occurrence of depression symptoms but not vice versa.

Cognitive disorders and depression have become a health problem, as there is an association of both transversely in the elderly, and early diagnosis could reduce other health problems, such as isolation, loneliness, and dependency. Depression often coexists with physical harm and social problems. It is essential to investigate the risk factors of cognitive impairment in our population with the goal of that older adults with cognitive impairment have a personalized and effective treatment to prevent the development of dementia. Patients with dementia have a poor prognosis, have an increased risk of institutionalization and are a major cause of stress on caregivers. For this reason, early detection of cognitive impairment may be beneficial for the patient, his family and the health sector.

The elderly population suffers a high incidence of disorders associated with cognitive impairment and depressive symptoms. For example, the incidence of depression secondary to Parkinson's disease has been estimated at around 40%, the prevalence of poststroke depressive syndrome between 30% and 50%, and the incidence of depression secondary to Alzheimer's disease from 10% to 20% [27].

Health personnel should also know that cognitive impairment has a high prevalence in settings as general hospitals. However, clinical experience suggests that these disorders often go unnoticed by medical nursing staff.

According to Delphi consensus study, in the United States and Canada, the incidence for dementia syndrome in people over 60 years was 6.4%. In Western Europe, a prevalence of 5.4% was reported. This consensus dementia prevalence was slightly higher than in Latin America with a 4.6% [28].

On the other hand, in the United States, depression alone represents a forty three billion dollar annual expense. Although the prevalence of depression may vary depending on the population studied and the methodology applied, its range is between 10% and 27% [29]. The prevalence of depression in the Canadian Community Health Survey: Mental Health and Well-Being (CCHS 1.2) was slightly lower than that reported in the US and comparable to Pan-European estimates [30].

The study of prevalence of cognitive impairment is essential because it constitutes an important risk factor for developing dementia syndrome. Although rates of progression are widely different across studies and populations, it has been found that the rate at which patients with cognitive impairment progress to dementia is about 70% over a period of 5 years, that is, 10%–15% per year as opposed to 1%-2% of control subjects [31]. Thus, its early detection is of great importance to develop preventive and early rehabilitation.

 Inferring causality in the relation between depression and cognitive impairment in old age has been hampered by the fact that most studies have examined only one direction of this relation. Some studies found that depression is a risk factor for the development of cognitive decline, whereas others could not confirm this finding. The relationship between depression and cognitive impairment shows that depression in old age is a concomitant phenomenon of already existing cognitive impairment rather than an independent risk factor. Our findings, based on various measures of cognitive function instead of a dichotomous end point, are in line with those from a large population-based study in people aged 65 and older showing that depression is an early manifestation rather than a predictor of Alzheimer's disease. Thus, in elderly people, the presence of depressive symptoms does not mean that they are at increased risk of cognitive decline.

## 5. Conclusions

Cognitive impairment and depression are highly correlated in adults aged 55 and more. Cognitive disorders and depression have become a health problem in developing countries.

## Figures and Tables

**Table 1:** Sociodemographic differences in 1142 adults studied (413 men and 729 women) from Guadalajara, Jalisco, Mexico: marital status, education, and social benefit of retired or pensioned.

	Female	Male	Total	*P*
	*n* (%)	*n* (%)	*n* (%)	
Sex	729 (63.8)	413 (36.2)	1141(100.0)	
Age				
60–69	362 (49.7)	179 (43.3)	541 (47.4)	0.123
70–79	234 (32.1)	155 (37.6)	389 (34.0)	
≥80	133 (18.2)	79 (19.1)	212 (18.6)	
Marital status				
Married	291 (39.9)	291 (70.5)	560 (49.0)	0.000
Not married	438 (60.1)	122 (29.5)	582 (51.0)	
Education (years)				
0	177 (24.3)	88 (21.3)	265 (23.2)	0.090
1–4	258 (35.4)	144 (34.9)	402 (35.2)	
5–8	185 (25.4)	104 (25.2)	289 (25.3)	
9–12	80 (11.0)	45 (10.9)	125 (10.9)	
≥13	29 (4.0)	32 (7.7)	61 (5.3)	
Retired or pensioned				
Yes	135 (18.5)	163 (39.5)	398 (26.1)	0.000
No	594 (81.5)	250 (60.5)	844 (73.9)	

**Table 2:** Prevalence of cognitive impairment and depression by gender (*n* = 1142).

	Cognitive impairment			Depression		
	Presence *n* (%)	CI 95%	Absence *n* (%)	Presence *n* (%)	CI 95%	Absence *n* (%)
Female	116 (14.5)	(12.1–17.4)	623 (85.5)	245 (33.6)	(30.2–37.2)	484 (66.4)
Male	52 (12.6)	( 9.6–16.3)	361 (87.4)	87 (21.1)	(17.3–25.4)	326 (78.9)

Total	158 (13.8)	(11.9–16.0)	984 (86.2)	332 (29.1)	(26.5–31.8)	810 (70.9)

**Table 3:** Relationship between cognitive impairment, sociodemographic data, and depression (*n* = 1142).

Variables	With cognitive impairment *n* (%)	Without cognitive impairment *n* (%)	OR crude	CI 95%	OR adjusted*	CI 95%
Gender						
Female	106 (14.5)	623 (85.5)	1.18	0.87–1.68	0.79	0.51–1.22
Male	52 (12.6)	361 (87.4)	1			
Age						
≥75	107 (26.7)	294 (73.3)	4.92	3.43–7.06	3.49	2.37–5.14
≤74	51 (6.9)	690 (93.1)	1			
Marital status						
Not married	117 (20.9)	443 (79.1)	3.48	2.39–5.08	2.73	1.77–4.19
Married	41 (7.0)	541 (93.0)	1			
Retired or pensioner						
No	126 (14.9)	718 (85.1)	1.45	0.96–2.20	1.24	0.77–1.99
Yes	32 (10.7)	266 (89.3)	1			
Education						
0–4	144 (21.6)	523 (78.4)	9.06	5.16–15.9	6.10	3.97–10.9
5–23	14 (2.9)	461 (97.1)	1			
Depression						
Yes	83 (25.0)	249 (75.0)	3.26	2.31–4.60	2.29	1.57–3.36
No	75 (9.3)	735 (90.7)	1			

OR: odd ratio.

*OR adjusted by gender, age, marital status, pensioner, education, and depression.

**Table 4:** Relationship between depression, sociodemographic data, and cognitive impairment (*n* = 1142).

Variables	With depression *n* (%)	Without depression *n* (%)	OR crude	CI 95%	OR adjusted	CI 95%
Gender						
Female	245 (33.6)	484 (66.4)	1.89	1.43–2.51	1.69	(1.24–2.32)
Male	87 (21.1)	326 (78.9)	1			
Age						
≥75	133 (33.2)	268 (66.8)	1.35	(1.03–1.76)	0.95	(0.68–1.23)
≤74	199 (26.9)	542 (73.1)	1			
Marital status						
Not married	200 (35.7)	360 (64.3)	1.89	(1.46–2.45)	1.42	(1.06–1.91)
Married	132 (22.7)	450 (77.3)	1			
Retired or pensioner						
No	126 (14.9)	718 (85.1)	1.45	(0.96–2.20)	1.01	(0.72–1.41)
Yes	32 (10.7)	266 (89.3)	1			
Education						
0–4	248 (37.2)	419 (62.8)	2.75	(2.07–3.65)	2.31	(1.70–3.13)
5–23	84 (17.7)	391 (82.3)	1			
Cognitive impairment						
Yes	83 (52.5)	75 (47.5)	3.26	(2.31–4.60)	2.33	(1.60–3.40)
No	249 (25.3)	735 (74.7)	1			

OR: odd ratio.

*OR adjusted by gender, age, marital status, pensioner, education, and cognitive impairment.
